# Futurization of Aging: Subjective Beliefs and Effects

**DOI:** 10.3390/bs13010004

**Published:** 2022-12-21

**Authors:** Olga Strizhitskaya, Marina Petrash, Daria Golubitskaya, Maria Kuzmina, Kristina Krupina, Anton Shchukin, Elena Engelgardt

**Affiliations:** Faculty of Psychology, Saint Petersburg State University, 199034 Saint Petersburg, Russia

**Keywords:** futurization of aging, construction of aging, subjective beliefs, reported performance, lifestyle resources, psychological resources

## Abstract

Aging in the face of an increasing population and growing life expectancy is considered one of the major demographic challenges in modern society. Previous research has revealed that quality of life in aging could significantly differ depending on the resources one possesses. However, little attention has been given to the mechanisms of formation of these resources and the role of intentionality. In the present study, we identified 22 strategies that favor a better life quality in aging and analyzed them from the perspective of subjective beliefs and reported performance. Our sample was adults (*n* = 72) aged 57–65, living in St. Petersburg, Russia. The results showed that although participants were aware of the strategies that favor aging, their reported performance ranged on a scale from average to infrequent use of these strategies. We found that subjective beliefs about the role of psychological resources for better aging predicted higher scores on subjective beliefs about the role of lifestyle resources and the reported performance of psychological resources. Our results suggest that there is a gap between subjective beliefs about the controllability of aging processes and the transformation of these beliefs into real performance.

## 1. Introduction

Over the last decades, increasing life expectancy and the related growth of the aging population have been seen as one of the greatest challenges of modern society [1]. Though life expectancy has been consistently growing, the quality of life of older adults has not directly followed this pattern. To approach the problem of maintaining and increasing the quality of life of older adults, scientists from different fields, including psychology, have investigated the nature, mechanisms, factors, and predictors of aging (for example, [1,2,3]). These studies have revealed plenty of mechanisms that affect aging, including, but not limited to, biological factors [4,5], lifestyle strategies [6,7], psychological mechanisms [8,9], and social environmental factors [10,11]. Most of these studies have focused on favorable or unfavorable effects for active/ healthy/successful aging. In 2019, the WHO, in its “Decade of Healthy Ageing 2020–2030”, reported the conceptualization of “healthy aging” as “the process of developing and maintaining the functional ability that enables wellbeing in older age” [12]. The key areas in which healthy aging is manifested, according to the WHO, are functional ability, intrinsic capacity, and environments. A systematic analysis of the studies conducted in the last twenty years showed [13] that resources of aging could be modified, and they differ in terms of the degree to which they can be modified. Whereas some resources, such as genetics, are hardly modifiable, environmental, lifestyle, and psychological resources could be more flexible for modifications. From this perspective, we can assume that if some resources of aging are modifiable, then a person can, to some extent, create their own aging process.

### 1.1. Futurization of Aging

The field of the futurization of thinking and behavior is relatively new. A. Sircova and her co-authors emphasize [14] that though futurization might have different connotations, for psychological research, the definition remains unclear, and for their own research, the authors focused on futurization as “an element of present behavior modification in light of a future scenario”.

Based on this definition, we suggest that this phenomenon could be applied to the processes of aging. We assume that many factors and mechanisms that are associated with positive outcomes in aging operate in a passive form: they are formed due to circumstances, but not following one’s intention. For example, cognitive reserve refers to a mechanism that is based on brain resources and strategies obtained during adulthood; it is the maintenance of cognitive abilities in aging [15]. It is formed when relevant cognitive activity occurs, regardless of one’s awareness that it is formed. At the same time, the activities that are relevant for the formation of cognitive reserve can be used intentionally or more frequently if one is aware of their effect and includes them in future scenarios. In this case, the formation of cognitive reserve may be transformed into a futurization of aging strategy.

### 1.2. Resources of Aging

Many biologists support the idea that there is no universal process of aging [4]; moreover, this process can be controlled much more strongly than many people used to think. Studies on genetic and environmental predictors of longevity and life expectancy have shown that genetic factors explain only 25–30% of the variance in life expectancy [4] and about 40% of the variance in longevity [16,17]. Though genetic information could be seen as hardly modifiable, its effects on the functioning of an organism can be moderated by environmental and social factors [16,18,19].

Environmental factors include a wide range of phenomena, from the economic status of one’s country and ecology to individual lifestyle. Thus, some environmental factors are controllable and modifiable, whereas others are not. Modifiable environmental factors include nutrition and diet [7,20,21], physical activity [6,22], sleep [23,24], and bad habits, such as smoking and alcohol consumption [25,26]. The positive effects of a healthy lifestyle on health and overall functioning in aging have been well-established. In particular, results have shown that nutrition and diet could affect cardiovascular health [27], moderate mortality from different causes [28], and influence physical and mental health [29,30]. The effects of physical activity for health and cognitive and psychological functioning have been shown in a range of studies [31,32]. Previous research has suggested that an increase in physical activity prevents cognitive declines [33] and the development of different forms of dementia, including Alzheimer’s disease [34,35]. One’s chronotype and schedule of sleep were found to affect life expectancy, longevity, cardiovascular health, and risk of diabetes and Alzhemer’s disease [23,24].

In the cognitive field, cognitive reserve can be seen as one of the key preventive concepts, as well as one of the most obvious examples of the futurization of aging [15]. By understanding which activities are most effective in maintaining cognitive functioning, we can incorporate such activities into our daily lives, reducing the likelihood of degenerative phenomena. Moreover, the range of possible activities is quite wide, which allows a person to choose their optimal types of activities.

Data on personality and emotional resources of aging are more complicated. On the one hand, research demonstrates the strengths of one’s emotional sphere and the personality of older adults, e.g., a positive paradox of aging, better emotional regulation [36,37]; and changes in personality towards maturity from the perspectives of both the Big Five model and Dark Triad model [38]. Further, we can assume that the results reported in the studies on older adults were formed throughout the adults’ lifespan. On the other hand, modern psychology does not give clear explanations of how these favorable outcomes are achieved. Finally, from a psychotherapeutic perspective, we could argue that though some positive outcomes could be formed in the face of one’s life situation without intention, a conscious modification of personality or emotional strategies constitutes outstandingly hard work. Thus, in light of the present work, we assumed that one’s personality and emotional resources of aging could be formed, but would need significant efforts.

The social sphere and, related to it, prosocial behavior have always been considered important factors for the wellbeing and mental and physical health of older adults (for example, [39,40]). Studies in this field have reported the role of prosocial behaviors in general [11], as well as in generativity [39], in one’s quality of personal relationships [41], and in volunteering [10]. The findings underlined the complexity of prosocial behavior and revealed that it was formed from early adulthood and even adolescence throughout the whole lifespan [11]. Although prosocial behavior seems to be natural for humans, associated with evolutionary processes and social approval, their development is moderated by a variety of factors. Thus, prosocial behavior could be stimulated to achieve better results and stronger outcomes for aging.

To summarize the previous research, the factors and mechanisms that have been shown to have a positive effect on quality of life, mental and physical health, and psychological wellbeing in aging are developed throughout the lifespan, but this development is not predetermined. Rather, it is based on genetic predispositions (physical, metabolic, cognitive, emotional, etc.), but it is activated and mediated by non-genetic factors, including one’s conscious intent. These conclusions give us ground to suggest that many of these resources could be developed by modification of one’s behavior with regard to the future benefits drawn from including simple strategies that develop resources for aging in a daily routine.

Our study aimed to approach processes related to the futurization of aging. We focused on resources that are associated with positive outcomes in aging in previous research, and we analyzed to what extent subjective beliefs about the impact of these strategies on aging are related to the performance of these strategies in the past. We hypothesized that awareness about the effects of lifestyle strategies in aging would be higher, since associations between a “healthy” lifestyle and health are well-established, and health has been considered one of the crucial domains for maintaining quality of life in aging [4,17]. We expected that those who reported higher scores on subjective beliefs about strategies of aging would also report higher scores on the performance of these strategies in the past. At the same time, we assumed that there might be a gap between subjective beliefs and reported performance, so we suggested that there might be no association between scores on subjective beliefs and real performance in the past. Finally, we hypothesized that if strategies of aging are supposed to have a positive impact on aging, they could be associated with psychological wellbeing not only in aging, but also during adulthood.

## 2. Materials and Methods

### 2.1. Data Collection and Research Design

This study was a part of a project entitled “Futurization of aging as a resource of maintaining quality of life in aging” and thus aimed at an analysis of the strategies and resources used by adults to create a better life through the aging process. The study was conducted in Saint Petersburg, Russia, from June 2022 to September 2022.

In our study, participants were older middle-aged adults (*n* = 72) aged 57–65 (M = 59.95, SD = 3.35, 33.3% males). A total of 72.2% had a university degree. Participants came from different professional backgrounds: education, medicine, management, accounting, engineering, etc. They had a similar income and reported to “have enough money for everyday life” (53%) and to “have enough money for everyday life and can afford vocation travel” (42%). Exclusion criteria were mental health issues (based on self-report) or cognitive decline (based on the results of cognitive tests that were part of the general study, but not included in the presented results).

Participants were recruited via the community (information was given at social meetings) and social networks. There was a preliminary talk at which the aims of the study were explained to participants, as well as its focus, main procedures, and their rights. The talk was performed in person or via conference means of communication. It was individual. No incentives were given to the participants. Questionnaires were distributed in person and via online forms (Google forms). Links to Google forms were sent only to participants who had completed the preliminary talk and given their consent to participate in the study. The research design, procedures, measures, and sampling were approved by the review board of the Russian Science Foundation (project No. 22-28-00869) and the Ethical Committee (IRB) of St. Petersburg Psychological Society (protocol No. 15, 22.05.2022). Informed consent was obtained from all participants, and the Helsinki declaration was respected.

Our research questions were:

(1) To what extent are people in the transition period between adulthood and aging aware of strategies that could help them to form resources for a better life in aging?
(2) How often were strategies of aging formation used by our participants 10 years ago?
(3) Could subjective beliefs affect the reported performance of the formation of strategies of aging?

We hypothesized that:

- Our study is exploratory, so we had no predictions about the extent to which our participants would be familiar with the formation of strategies of aging, but we expected that those who were more familiar would demonstrate higher scores on reported performance as well.
- Transferring the idea that more knowledge about aging in general, and particularly about stereotypes of aging, leads to better self-understanding and better mental functioning [40], we supposed that more knowledge about the formation of strategies of aging would also lead to a higher use of these strategies.

### 2.2. Measures

Our study concentrated on the idea that resources that determine quality of life in aging could be formed at earlier lifespan stages. Based on a systematic literature review of research conducted in the last 20 years [13], we identified 22 strategies that showed positive outcomes for quality of life in aging. These strategies were:

(1) control over different types of fats (based on consultations with doctors);
(2) going in for sports or fitness;
(3) continued learning;
(4) participation in social events;
(5) the ability to not be nervous about minor negative events;
(6) the ability to not think about problems when going to sleep;
(7) intentional control over one’s sleep schedule;
(8) intentional refusal to use gadgets before going to sleep;
(9) following a healthy diet;
(10) watching one’s water balance;
(11) intention to solve complex problems (brain activity);
(12) reading;
(13) learning foreign languages;
(14) acquiring new impressions;
(15) driving;
(16) thinking about how one’s own memory, attention, and cognitive abilities work;
(17) thinking about how different processes (mental, emotional, physiological) work;
(18) striving to regulate one’s own emotions;
(19) self-analysis;
(20) generativity;
(21) a desire to help others;
(22) control of one’s quality of sleep.

We used this list of strategies with two instructions: (1) we asked participants to estimate on a Likert scale from 1 to 5 how often they practiced these strategies 10 years ago, where 1 would be “never did it”, 5—used this strategy regularly (reported performance—RP); (2) we asked participants to estimate on a Likert scale from 1 to 5 whether they thought that these strategies affected positive aging (subjective beliefs—SB). These two tasks were separated by several different questionnaires to create a small interference effect.

For the purposes of the present study, we explored the internal structure of this list using exploratory factor analysis (EFA). We identified two subscales, each of which included 11 strategies. The first subscale included strategies related to a healthy lifestyle (1, 2, 5, 6, 7, 8, 9, 10, 12, 18, 22), and we called it “Lifestyle resources (LR)”. The second factor included strategies that were more related to psychological processes (3, 4, 11, 13, 14, 15, 16, 17, 19, 20, 21), and we called it “Psychological resources (PR)”. Based on these results, we computed new variables for both instructions: LR and PR for strategies used 10 years ago, and LR and PR for beliefs about the impact of these strategies on positive aging. In present paper, we used a strategies score and a subscale score for different analyses.

### 2.3. Statistical Analysis

Data were processed using the statistical program Statistical Package for Social Sciences SPSS (v20.0, SPSS Inc., Chicago, IL, USA). We used descriptive statistics, correlation analysis (Pearson r-coefficient), exploratory factor analysis (EFA) using the alpha-factorization method, and alpha-Cronbach analysis. Where applicable, we used Bootstrap procedures to confirm the significance of the results.

## 3. Results

To address Research Question 1, we analyzed descriptive statistics for subjective beliefs about strategies of aging formation (SB-SAF) and reported the performance of the formation of strategies of aging in the past (RP-SAF) (Table 1). The results showed that for both psychological and lifestyle strategies, SAF mean scores for subjective beliefs were higher than for reported performance. This result suggested that overall, the awareness of activities that favor a better life in aging was relatively high in our sample. At the same time, the reported performance of SAF ranged from average to low. Overall, our results revealed that our participants reported higher scores on the reported performance of SAF in the psychological domain than in the lifestyle domain, although they allotted comparatively similar importance to these strategies in terms of their impact on healthy aging. Correlation analysis revealed that 9 out of 11 SAF in the domain of psychological resources were correlated, whereas for the lifestyle domain, we found only four correlations.

Differential analysis of strategies of aging formation (SAF) showed that for subjective beliefs about lifestyle resources, the top-rated strategies were “Following healthy diet” (M = 4.35), “Watching water balance” (M = 4.21), and “Control of quality of sleep” (M = 4.48). The lowest-rated strategy was “Controlling fats” (M = 3.27). We supposed that the top-rated strategies could reflect not only the extent of the awareness about the role of these resources, but also the “problem” zones that attracted our participants’ attention. “Controlling fats” is quite a new direction in healthy lifestyle studies [18]. This strategy includes the need for knowledge about the variety of fats and their differential impact on one’s health. This information would require more scientific research, rather than being available via general mass media channels. As such, we suggest that low scores could reflect low awareness about this strategy.

In the domain of psychological resources, the top-rated strategies were “Intention to solve complex problems” (M = 4.24), “Acquiring new impressions” (M = 4.35), and “Desire to help others” (M = 4.24). The lowest-rated strategy was “Continuing learning” (M = 3.27). These results refer to three main psychological domains: the cognitive, emotional, and social components of healthy aging, and two of them—“Acquiring new impressions” and “Desire to help others”—also demonstrate the value that participants gave to strategies related to wellbeing (impressions are associated with personal growth, helping others—with positive relations with others). Interestingly, our participants underestimated the role of “Continuing learning”, although it has been well-established in society.

To address Research Question 2, we analyzed the reported performance and found that most strategies from both domains were used “from time to time” (around a score of “3”). Nevertheless, the “Desire to help others” appeared on the scale from “often” to “regularly”, and “controlling fats” was used “seldom”. This result could be attributed to two tendencies: (1) “helping others” could be a socially appreciated characteristic, and (2) “controlling fats” was not so widely known.

**Figure 1:**
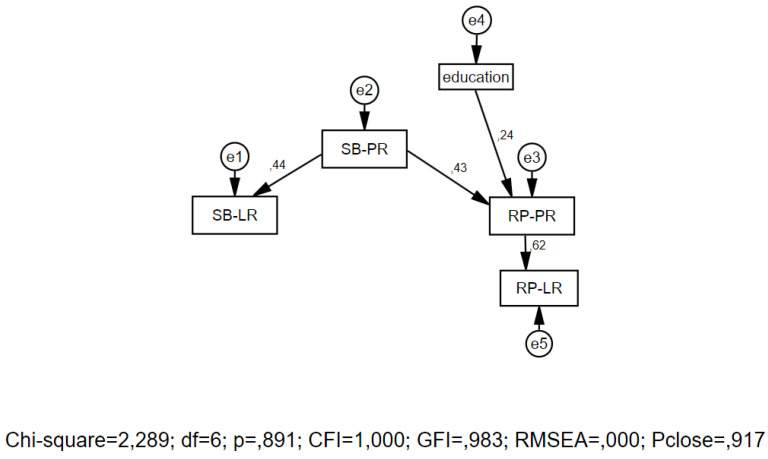
Path model of composite variables for subjective beliefs and reported performance. SB-LR—subjective beliefs—lifestyle resources, SB-PR—subjective beliefs—psychological resources, RP-LR real-time performance—lifestyle resources, RP-PR—real-time performance—psychological resources.

To address Research Question 3, we conducted a regression path analysis using AMOS 20.0 (Figure 1). For this analysis, we used the composite variables for subjective beliefs and reported performance for both lifestyle and psychological resources. Composite variables were based on EFA, which was applied to the list of strategies of aging formation (subjective beliefs). EFA revealed a Kaiser–Meyer–Olkin Measure of Sampling Adequacy of 0.788, and variance explained 43.57% (22.12% for lifestyle resources and 21.45% for psychological resources). Next, we ran scale reliability tests using the alpha-Cronbach score, which was 0.882 for LR and 0.862 for PR.

We also used the demographic characteristic of “education”, because we supposed that this could affect the domain of psychological resources. The analysis showed that subjective beliefs about the role of psychological resources predicted both subjective beliefs about lifestyle resources and the reported performance of psychological resources. The reported performance of psychological resources, in turn, predicted the reported performance of lifestyle resources. We also found that education affected the reported performance of psychological resources, but not subjective beliefs. Our results showed that all standardized estimates were positive. Any changes in the directions of paths caused decreases in model fit indexes.

## 4. Discussion

In the present paper, we have made an attempt to approach the complicated problem of controllability and modifiability in the process of aging. Based on systematic analysis, we identified 22 strategies that helped the formation of resources for better aging and asked adults who were in the period of transition from adulthood to aging (57–65) to report their beliefs and performance of those strategies.

The present study contributes to the understanding of healthy aging by expanding the understanding of healthy aging as presented by the WHO [12] to a broader lifespan view. The WHO underlines that aging is a *process*, but our contribution is that this process starts long before the aging period as it is usually seen (65+). Futurization of aging suggests that knowledge about the possibilities of the construction of aging might help people to incorporate a variety of strategies and mechanisms (for example, [5,9,10,17]) in their daily routine for a better later life.

Our results showed relatively high awareness about the strategies of aging among our participants. Interestingly, we found that participants showed the same level of awareness about psychological and lifestyle resources. We expected that participants would be aware of healthy lifestyle habits, as the overall societal focus on health is quite high and is promoted by medical institutions. Still, we did not have clear predictions about the awareness of the role of psychological resources for aging among adults, since this topic has received much less attention in our society. We also found that the reported performance of these strategies was average, sometimes even low. We suggest several interpretations for this fact. First, we asked participants to report how often they used these strategies 10 years ago, which means that 10 years ago, they might not know about these strategies. Second, we speculated that subjective beliefs referred to mental representations of some knowledge, whereas real performance needed to transform a mental representation into a behavioral act: going to the gym, calculating calories, participating in a volunteering project, etc. Thus, further research is needed to address mechanisms that promote or prevent such a transformation.

Our results also showed that subjective beliefs about the role of SAF in the psychological domain predicted subjective beliefs about lifestyle strategies and the reported performance of psychological strategies. This leading role of subjective beliefs about psychological resources could open a new understanding of the aging construction: we could hypothesize that those who believe that psychological regulation (self-analysis, understanding of one’s mental abilities, and regulation of relationships with others) can help to form their aging could also believe that their very aging might be more controllable. If so, starting from the idea that one can control their own aging, he/she might move to a broader context of modifications of their own resources and behavior. Supporting this idea, we found that education also promoted higher scores on reported performance in the psychological domain. This idea was also consistent with the results, showing that a better understanding of the specifics of aging helped to create better functioning in aging [42].

Our study has several limitations. First, it used a relatively small sample size that was sufficient for conducting all analyses presented in the paper, but with this sample, we were not able to control for demographic covariates. Future research needs to address the effects of such factors as sex, education, and professional trajectories. Second, our study used a cross-sectional design, so future research is needed to reveal the longitudinal dynamics of the formation of aging. Finally, in the present study, we focused on the strategies themselves, but we expect that their functioning could be moderated or mediated by psychological or demographic variables.

In future research, we need to increase the sample, particularly the age range, so that there might be a chance to estimate at what point an “investment” in healthy aging is most powerful. Future research also needs to use qualitative methods together with quantitative ones for a better understanding of the mechanisms of the futurization of aging and the factors preventing people from creating their own aging process. Finally, longitudinal studies are needed to prove the dynamics of the futurization of aging and to create prognostics models of such futurization.

## Figures and Tables

**Table 1:** Descriptive statistics and correlations of strategies of aging formation.

Strategies of Aging Formation (SAF)	SB-SAF		RP-SAF		*Cor.*	*p*	Bootstrap CI (95%)	
	M	SD	M	SD			Low Limit	High Limit
Psychological resources								
Continued learning	3.27	1.10	3.29	1.34	−0.126	0.312	−0.484	0.409
Participation in social events	3.39	1.19	3.11	1.49	0.393	**0.001**	−0.091	0.636
Intention to solve complex problems	4.24	0.86	3.27	1.20	0.189	0.129	0.652	1.258
Learning foreign languages	3.47	1.38	2.17	1.40	0.269	**0.029**	0.864	1.697
Acquiring new impressions	4.35	0.77	3.52	1.30	0.398	**0.001**	0.530	1.167
Driving	3.41	1.45	2.94	1.82	0.529	**0.000**	0.061	0.833
Thinking about how own memory, attention, cognitive abilities work	3.52	1.10	3.00	1.44	0.380	**0.002**	0.167	0.879
Thinking about how different processes work	3.52	1.13	2.59	1.40	0.291	**0.018**	0.545	1.287
Self-analysis	3.45	1.15	3.08	1.35	0.343	**0.005**	0.016	0.727
Generativity	3.91	0.99	3.53	1.29	0.400	**0.001**	0.061	0.682
Desire to help others	4.24	0.90	4.39	0.74	0.456	**0.000**	−0.364	0.061
Lifestyle resources								
Controlling fats	3.27	1.38	1.67	1.00	−0.022	0.858	1.197	2.015
Going in for sports or fitness	3.71	1.15	2.61	1.35	0.025	0.842	0.652	1.530
Not being nervous about minor negative events	4.12	0.95	3.05	1.23	−0.096	0.441	0.652	1.484
Not thinking about problems when going to sleep	4.12	0.94	2.91	1.20	−0.031	0.804	0.803	1.576
Intentional control over sleep schedule	4.14	1.01	2.73	1.38	0.216	0.081	1.061	1.757
Intentional refusal to use gadgets before going to sleep	3.98	0.98	2.47	1.32	0.291	**0.018**	1.152	1.848
Following healthy diet	4.35	0.89	2.89	1.25	−0.119	0.343	1.076	1.818
Watching water balance	4.21	0.90	2.79	1.20	0.213	0.086	1.106	1.727
Reading	4.05	1.09	3.35	1.20	0.319	**0.009**	0.394	1.015
Striving to regulate own emotions	3.95	0.98	3.00	1.24	0.441	**0.000**	0.667	1.258
Controlled of quality of sleep	4.48	0.73	3.17	1.21	0.291	**0.018**	1.030	1.636

## Data Availability

The data presented in this study are available on request from the corresponding author.

## References

[1] Rowe J.W., Kahn R.L. (2015). Successful Aging 2.0: Conceptual Expansions for the 21st Century. J. Gerontol. Ser. B Psychol. Sci. Soc. Sci..

[2] Baltes P.B., Baltes M.M., Baltes P.B., Baltes M.M. (1990). Psychological Perspectives on Successful Aging: The Model of Selective Optimization with Compensation. Successful Aging: Perspectives from the Behavioral Sciences.

[3] Olshansky S.J., Perry D., Miller R.A., Butler R.N. (2007). Pursuing the Longevity Dividend. Ann. N. Y. Acad. Sci..

[4] Kirkwood T.B.L. (2017). Why and how are we living longer?. Exp. Physiol..

[5] Fong B.Y.F., Chiu W.-K., Chan W.F.M., Lam T.Y. (2021). A review study of a green diet and healthy ageing. Int. J. Environ. Res. Public Health.

[6] Stessman J., Jacobs J.M. (2014). Diabetes Mellitus, Physical Activity, and Longevity Between the Ages of 70 and 90. J. Am. Geriatr. Soc..

[7] Kuzuya M. (2021). Nutritional status related to poor health outcomes in older people: Which is better, obese or lean?. Geriatr. Gerontol. Int..

[8] Harris K., English T., Harms P.D., Gross J.J., Jackson J.J. (2017). Why are Extraverts more satisfied? Personality, Social Experiences, And Subjective Well-Being In College. Eur. J. Personal..

[9] Potter S., Drewelies J., Wagner J., Duezel S., Brose A., Demuth I., Steinhagen-Thiessen E., Lindenberger U., Wagner G.G., Gerstorf D. (2020). Trajectories of multiple subjective well-being facets across old age: The role of health and personality. Psychol. Aging.

[10] Bjälkebring P., Henning G., Västfjäll D., Dickert S., Brehmer Y., Buratti S., Hansson I., Johansson B. (2021). Helping Out or Helping Yourself? Volunteering and Life Satisfaction Across the Retirement Transition. Psychol. Aging.

[11] Shane J., Niwa E.Y., Heckhausen J. (2021). Prosociality Across Adulthood: A Developmental and Motivational Perspective. Psychol. Aging.

[12] World Health Organization: Decade of Healthy Ageing 2020–2030” reported conceptualized. https://www.who.int/docs/default-source/documents/decade-of-health-ageing/decade-healthy-ageing-update-march-2019.pdf?sfvrsn=5a6d0e5c_2.

[13] Strizhitskaya O.Y., Petrash M.D. (2022). Construction of Productive Ageing: Biological, Psychological and Environmental Factors [Elektronnyi resurs]. Konsul’tativnaya Psikhologiya I Psikhoterapiya Couns. Psychol. Psychother..

[14] Sircova A., Oliveira L. (2019). Futurization of Thinking and Behavior: Exploring People’s Imaginaries About the Future and Futurization. Managing Screen Time in an Online Society.

[15] Nelson M.E., Jester D.J., Petkus A.J., Andel R. (2021). Cognitive Reserve, Alzheimer’s Neuropathology, and Risk of Dementia: A Systematic Review and Meta-Analysis. Neuropsychol. Rev..

[16] Häsler R., Venkatesh G., Tan Q., Flachsbart F., Sinha A., Rosenstiel P., Lieb W., Schreiber S., Christensen K., Christiansen L. (2017). Genetic interplay between human longevity and metabolic pathways—A large-scale eQTL study. Aging Cell.

[17] Murabito J.M., Yuan R., Lunetta K.L. (2021). The search for longevity and healthy aging genes: Insights from epidemiological studies and samples of long-lived individuals. J. Gerontol. Ser. A Biol. Sci. Med. Sci..

[18] Schroeder E.A., Brunet A. (2015). Lipid Profiles and Signals for Long Life. Trends Endocrinol. Metab. TEM.

[19] Zarulli V., Barthold Jones J.A., Oksuzyan A., Lindahl-Jacobsen R., Christensen K., Vaupel J.W. (2018). Women live longer than men even during severe famines and epidemics. Proc. Natl. Acad. Sci. USA.

[20] Jacobs J.M., Cohen A., Ein-Mor E., Stessman J. (2013). Cholesterol, statins, and longevity from age 70 to 90 years. J. Am. Med. Dir. Assoc..

[21] Samieri C., Jutand M.A., Feart C., Capuron L., Letenneur L., Barberger-Gateau P. (2008). Dietary patterns derived by hybrid clus-tering method in older people: Association with cognition, mood, and self-rated health. J. Am. Diet. Assoc..

[22] Fortes C., Mastroeni S., Sperati A., Pacifici R., Zuccaro P., Francesco F., Ebrahim S. (2013). Walking four times weekly for at least 15min is associated with longevity in a Cohort of very elderly people. Maturitas.

[23] Cullell N., Cárcel-Márquez J., Gallego-Fábrega C., Muiño E., Llucià-Carol L., Lledós M., Amaut K.E.U., Krupinski J., Fer-nández-Cadenas I. (2021). Sleep/wake cycle alterations as a cause of neurodegenerative diseases: A Mendelian randomization study. Neurobiol. Aging.

[24] Didikoglu A., Maharani A., Payton A., Pendleton N., Canal M.M. (2019). Longitudinal change of sleep timing: Association between chronotype and longevity in older adults. Chronobiol. Int..

[25] Zanjani F., Downer B.G., Kruger T.M., Willis S.L., Schaie K.W. (2013). Alcohol effects on cognitive change in middle-aged and older adults. Aging Ment. Health.

[26] Arntzen K.A., Schirmer H., Wilsgaard T., Mathiesen E.B. (2010). Moderate wine consumption is associated with better cognitive test results: A 7 year follow up of 5033 subjects in the Tromsø Study. Acta Neurol. Scand..

[27] McNaughton S.A., Dunstan D.W., Ball K., Shaw J., Crawford D. (2009). Dietary quality is associated with diabetes and car-diometabolic risk factors. J. Nutr..

[28] McNaughton S.A., Bates C.J., Mishra G.D. (2012). Diet quality is associated with all-cause mortality in adults aged 65 years and older. J. Nutr..

[29] Bourre J.M. (2006). Effects of nutrients (in food) on the structure and function of the nervous system: Update on dietary requirements for brain. Part 2: Macronutrients. J. Nutr. Health Aging.

[30] Bourre J.M. (2006). Effects of nutrients (in food) on the structure and function of the nervous system: Update on dietary requirements for brain. Part 1: Micronutrients. J. Nutr. Health Aging.

[31] Paterson D.H., Warburton D.E. (2010). Physical activity and functional limitations in older adults: A systematic review related to Canada’s physical activity guidelines. Int. J. Behav. Nutr. Phys. Act..

[32] Renner B., Spivak Y., Kwon S., Schwarzer R. (2007). Does age make a difference? Predicting physical activity of South Koreans. Psychol. Aging.

[33] Simons L.A., Simons J., McCallum J., Friedlander Y. (2006). Lifestyle factors and risk of dementia: Dubbo study of the elderly. Med. J. Aust..

[34] Miller D.I., Taler V., Davidson P.S., Messier C. (2012). Measuring the impact of exercise on cognitive aging: Methodological issues. Neurobiol. Aging.

[35] Shubert T.E., McCulloch K., Hartman M., Giuliani C.A. (2010). The effect of an exercise-based balance intervention on physical and cognitive performance for older adults: A pilot study. J. Geriatr. Phys. Ther..

[36] Mather M. (2012). The emotion paradox in the aging brain. Ann. N. Y. Acad. Sci..

[37] Scheibe S., Carstensen L.L. (2010). Emotional Aging: Recent Findings and Future Trends. J. Gerontol. Ser. B Psychol. Sci. Soc. Sci..

[38] Kawamoto T., Shimotsukasa T., Oshio A. (2020). Cross-sectional age differences in the Dark Triad traits in two Japanese samples. Psychol. Aging.

[39] Sparrow E.P., Swirsky L.T., Kudus F., Spaniol J. (2021). Aging and Altruism: A Meta-Analysis. Psychol. Aging.

[40] Townsend B.G., Chen J.T.-H., Wuthrich V.M. (2021). Barriers and Facilitators to Social Participation in Older Adults: A Systematic Literature Review. Clin. Gerontol..

[41] Nikitin J., Freund A.M. (2021). Does Focusing on Others Enhance Subjective Well-Being? The Role of Age, Motivation, and Rela-tionship Closeness. Psychol. Aging.

[42] Levy B.R., Chang E.-S., Lowe S.R., Provolo N., Slade M.D. (2022). Impact of Media-Based Negative and Positive Age Stereotypes on Older Individuals’ Mental Health. J. Gerontol. Ser. B Psychol. Sci. Soc. Sci..

